# Integrated MicroRNA and mRNA Signatures Associated with Survival in Triple Negative Breast Cancer

**DOI:** 10.1371/journal.pone.0055910

**Published:** 2013-02-06

**Authors:** Luciano Cascione, Pierluigi Gasparini, Francesca Lovat, Stefania Carasi, Alfredo Pulvirenti, Alfredo Ferro, Hansjuerg Alder, Gang He, Andrea Vecchione, Carlo M. Croce, Charles L. Shapiro, Kay Huebner

**Affiliations:** 1 Department of Molecular Virology, Immunology and Medical Genetics, Ohio State University Wexner Medical Center and Comprehensive Cancer Center, Columbus, Ohio, United States of America; 2 Department of Clinical and Molecular Biomedicine, University of Catania, Catania, Italy; 3 Department of Pathology, Ohio State University Wexner Medical Center, Division of Pathology II, Columbus, Ohio, United States of America; 4 University of Rome “La Sapienza”, Ospedale Santo Andrea, Rome, Italy; 5 Division of Medical Oncology and the Breast Program James Cancer Hospital and Ohio State University Comprehensive Cancer Center, Columbus, Ohio, United States of America; Sudbury Regional Hospital, Canada

## Abstract

Triple negative breast cancer (TNBC) is a heterogeneous disease at the molecular, pathologic and clinical levels. To stratify TNBCs, we determined microRNA (miRNA) expression profiles, as well as expression profiles of a cancer-focused mRNA panel, in tumor, adjacent non-tumor (normal) and lymph node metastatic lesion (mets) tissues, from 173 women with TNBCs; we linked specific miRNA signatures to patient survival and used miRNA/mRNA anti-correlations to identify clinically and genetically different TNBC subclasses. We also assessed miRNA signatures as potential regulators of TNBC subclass-specific gene expression networks defined by expression of canonical signal pathways.

Tissue specific miRNAs and mRNAs were identified for normal *vs* tumor *vs* mets comparisons. miRNA signatures correlated with prognosis were identified and predicted anti-correlated targets within the mRNA profile were defined. Two miRNA signatures (miR-16, 155, 125b, 374a and miR-16, 125b, 374a, 374b, 421, 655, 497) predictive of overall survival (P = 0.05) and distant-disease free survival (P = 0.009), respectively, were identified for patients 50 yrs of age or younger. By multivariate analysis the risk signatures were independent predictors for overall survival and distant-disease free survival. mRNA expression profiling, using the cancer-focused mRNA panel, resulted in clustering of TNBCs into 4 molecular subclasses with different expression signatures anti-correlated with the prognostic miRNAs.

Our findings suggest that miRNAs play a key role in triple negative breast cancer through their ability to regulate fundamental pathways such as: cellular growth and proliferation, cellular movement and migration, Extra Cellular Matrix degradation. The results define miRNA expression signatures that characterize and contribute to the phenotypic diversity of TNBC and its metastasis.

## Introduction

miRNAs are small (19–25 nucleotides), non-coding RNAs that reduce the abundance and translational efficiency of mRNAs and play a major role in regulatory networks, influencing diverse biological processes [1], [2] through effects of individual miRNAs on translation of multiple mRNAs. Determining roles of individual miRNAs in cellular regulatory processes poses a major challenge, with function of the majority of miRNAs currently unknown; even for relatively well-studied miRNAs, only a handful of targets have been rigorously characterized and these may differ by tissue type.

Triple-negative breast cancers are defined by a lack of expression of estrogen receptor (ESR1), progesterone receptor (PGR1), and ERBB2 receptor. This subgroup accounts for 15% of all types of breast cancer and is an aggressive form with limited treatment options.

We have used the nanoString nCounter platform (Seattle, WA, USA) to profile both miRNA and mRNA expression (nanoString Cancer Reference panel) using the same RNA sample from each breast cancer patient. Our analyses confirmed some observations from previous studies [3], [4] and revealed new specific miRNA signatures as potential biomarkers for distant-disease free (DDFS) and overall survival (OS). We emphasized the joint analysis of miRNA and mRNA data, and thoroughly analyzed anti-correlations between miRNA and mRNA expression data.

We also assessed, through target prediction analysis, if the miRNA signatures can potentially regulate and thus define pivotal pathways in mRNA defined TNBC subclasses. Moreover we were able to correlate tissue specific miRNA expression signatures, defined by comparisons among normal, tumor and metastatic tissues, with specific dysregulated mRNAs in the same comparisons. The analyses confirm the heterogeneity of TNBC and provide a basis for further molecular studies to develop miRNA-based early detection markers and novel therapeutic targets for triple negative breast cancer.

## Results

### miRNA expression profiles differentiate among adjacent normal breast and TNBC tissues

The profiles of 224 samples, 165 primary cancer-derived RNAs and 59 normal RNAs from patients with a median age of 51 years were considered. Hierarchical clustering represented in the heat map in Fig. S1, shows 116 miRNAs differentially expressed in TNBCs *vs* normal breast expression profiles, some with known involvement in breast cancer progression [5]. All fold-changes associated with these analyses are represented in log2 scale (logFC) and we show all data with a P-value of <0.05, considered to indicate statistical significance.

miR-106b [6] is over-expressed in tumor compared to normal (logFC 3.6), the Myc-regulated miR-17/92 oncomir cluster that characterizes TNBC [7] is strongly deregulated (miR-17, logFC 1.23; miR-106a, logFC 1.23; miR-20a, logFC 5.88; miR-20b, logFC 5.88; miR-19b, logFC 1.86). Members of the miR-8 family [8] are up-regulated: miR-200a (logFC 0.33), miR-200b (logFC 1.47), miR-200c (logFC 1.22). Two members of the let-7 family: let-7b (logFC −0.47) and let-7c (logFC −1.92) are down-regulated, as reported previously [9]. Tumor suppressor miRNAs down-regulated in tumor profiles are: miR-126 (logFC −0.74), involved in cell cycle progression and metastasis suppression [10]; miR-145 (logFC −2.83) associated with p53-mediated repression of Myc and suppression of cell invasion [11]; miR-205 (logFC −2.49), targeting ErbB3 and VEGF-A, and inhibiting tumor growth and invasion [12]. Other miRNAs, with oncogene activity, are up-regulated in our profiles: miR-21 (logFC 2.29) plays a crucial role in tumor cell proliferation, apoptosis, invasion, consistent with ability to repress tumor suppressors PTEN, PDCD4, TPM-1 [13] and SPRY2 [14]; miR-155 (logFC 2.68), −9 (logFC 3.13) and −107 (logFC 2.32) implicated in tumor aggressiveness and resistance to chemotherapy *in vitro* and *in vivo* [15].

Hierarchical clustering of the subset of 55 matched tumor and normal tissue RNAs is shown in Fig. S2; 104 miRNAs appear to be dysregulated. We still observe up-regulation of miR-106b (logFC 3.29), 17–92 cluster (average cluster deregulation, logFC 3.19), miR-9 (logFC 3.22), miR-21 (logFC 2.54) and miR-27a (logFC 1.21). In this matched cluster analysis, we found 33 miRNAs that were not appreciably altered in expression in the Fig. S1 heat map, with greatest fold changes in: miR-193a-3p (logFC −1.4), miR-19a (logFC 1.75) and miR-210 (logFC 1.64) (Table S1 A, B).

Several miRNAs have been shown to be involved in breast metastasis induction and progression, through processes such as epithelial-mesenchymal transition (EMT), extracellular matrix modification (ECM) and mesenchymal-epithelial transition (MET) [16]. In our study, RNAs of 54 TNBC-associated regional lymph node metastases (mets) were compared to the 59 normal RNAs, with metastatic miRNA profiles clustering distinctly separately from the normal profiles. The heat map in Fig. S3 shows 103 miRNAs (Table S2) deregulated in the normal *vs* mets comparison in the entire cohort (median age 51 yrs, range 20–84). We further investigated the deregulation of miRNAs in the three different tissue classes.

### Summary of dysregulated miRNAs among the tissue classes

**Figure 1:**
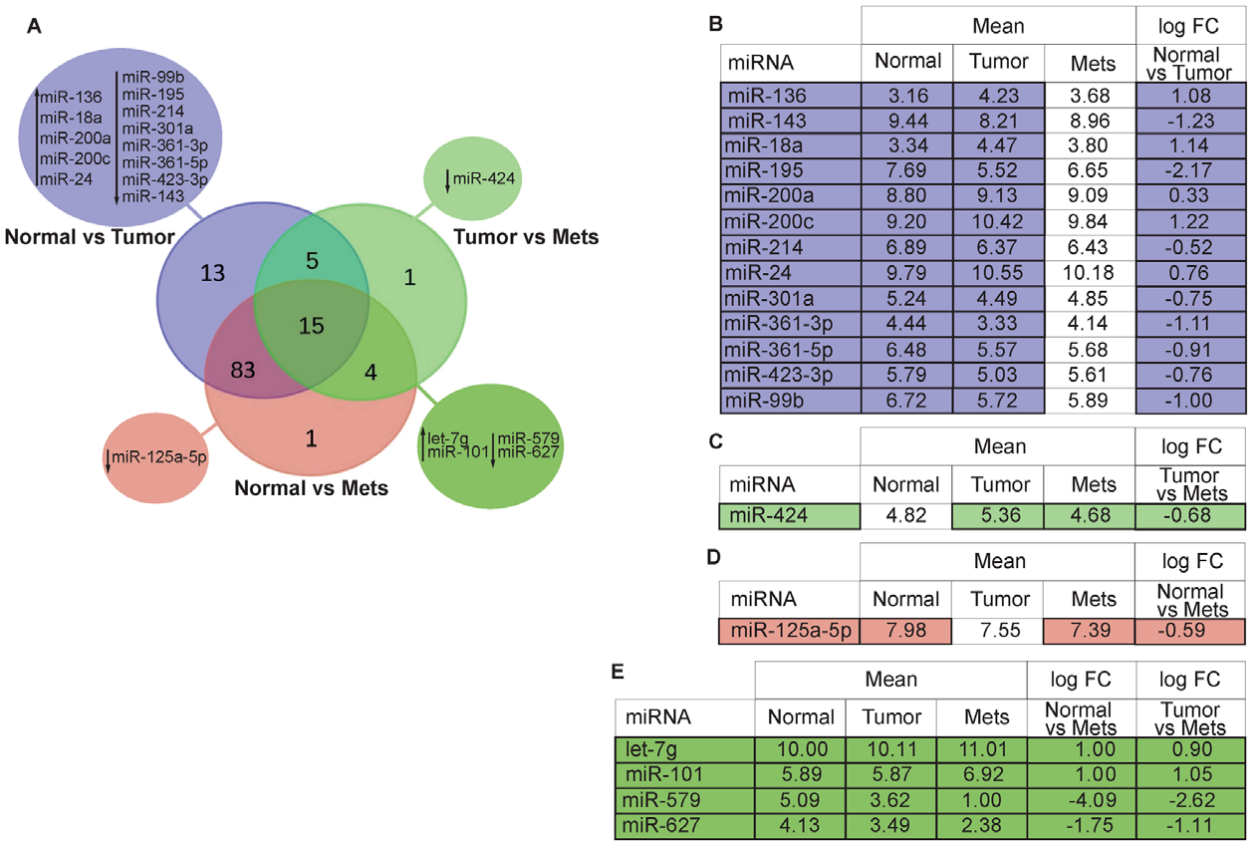
Venn diagram describing miRNA expression patterns in the three classes of breast tissue. Venn diagram (**A**) representing differentially expressed miRNAs observed in the comparisons among the three classes (normal, primary tumor, metastatic lesion), up or down facing arrow indicate the expression level change. Tables show: the 13 miRNAs identified by the comparison between Normal *vs* Tumor (**B**); 1 miR differentially expressed only in Tumor vs Mets (**C**); 1 miR differentially expressed only in Normal vs Mets (**D**); 4 miRNAs commonly deregulated in the Tumor vs Mets and Normal vs Mets comparisons (**E**).

The Venn diagram (Fig. 1) summarizes the number of differentially expressed miRNAs in the three tissue classes. 13 miRNAs (Fig. 1 B) represent the expression pathways dysregulated as cells progress from normal to primary TNBC (not significantly dysregulated in the other comparisons). Among these tumor specific deregulated miRNAs, members of the miR-8 family and miR-24 are strongly up-regulated, confirming previous findings [17]. We considered as a “metastatic signature” a pool of 6 miRNAs differentially modulated in tumor to metastatic and normal to metastatic transition but not in normal *vs* tumor (Fig. 1 C, D, E). The down-regulation of miR-424 distinguishes the tumor *vs* mets (logFC −0.68) comparison (Fig. 1 C); miR-125a-5p (logFC −0.59), distinguishes the normal *vs* mets comparison (Fig. 1 D), with down-regulation in the mets confirming it as a tumor suppressive miRNA involved in modulation of anchorage dependent cell growth [5]. Up-regulated in both these comparisons are let-7g (logFC 0.90, 1.01 respectively) and miR-101 (logFC 1.05, 1.03), miR-627 (logFC −1.11, −1.75), miR-579 (logFC −2.62, −4.09) are down-regulated. Table S3 lists the 15 miRNAs differentially expressed across the three tissue group comparisons, shown by the intersection of the 3 expression profiles in the central part of the Venn diagram in Fig. 1A, characterized by several miRNAs with high logFC in normal *vs* tumor *vs* mets comparisons. miR-542-3p (logFC −1.06, −2.65, −3.73, respectively, in normal>tumor>mets) is highly down-regulated through the three tissue classes; this is also in accordance with the anti-correlated trend, shown below in the mRNA profile (logFC 1.9, 1.7, 0.2), for its validated target *BIRC5* [18], an established anti-apoptotic gene. miR-125b (logFC −2.02, −0.73, −2.76) is down-regulated through the three tissue classes and mostly anti-correlated with its validated target, *CDKN2A*, which is up-regulated in our mRNA profile (logFC 2.6, 1.5, −1.1).

Examples of up-regulated miRNAs through the three tissue classes are: miR-128 (logFC 1.51, 1.15, 2.66) targeting *WEE1* [19], an important cell-cycle checkpoint regulator in breast cancer which in our profile shows an anti-correlated trend (logFC −1.6, −0.9, 0.7), similarly for miR-26b (logFC 2.27, 1.4, 3.66) and its validated target *TGFBR2* (logFC −2.3, −1.7, 0.6).

### miRNA expression signatures associated with survival

**Figure 2:**
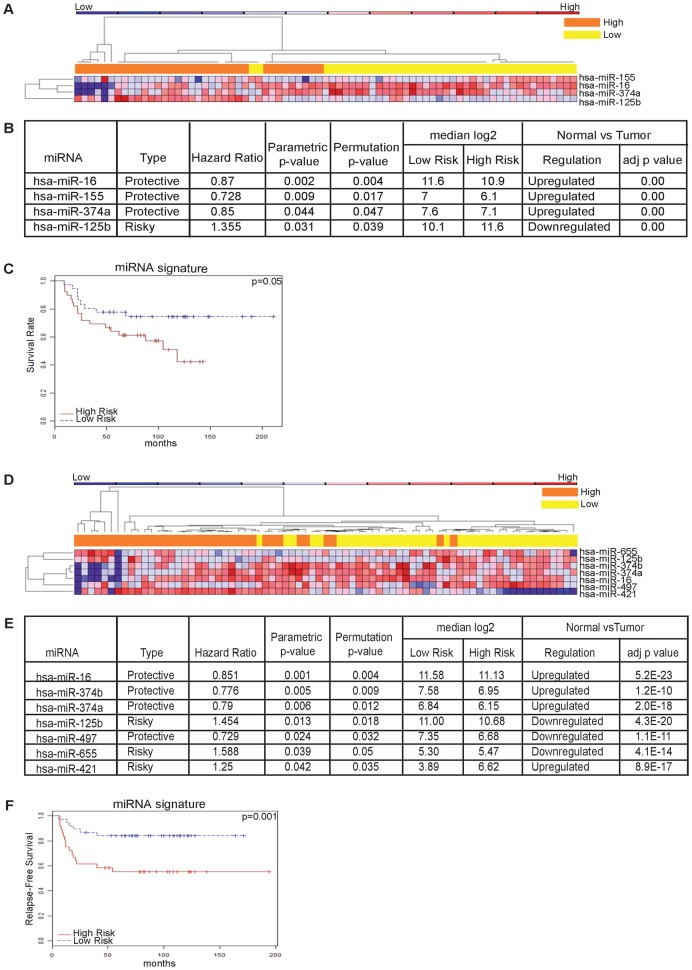
Overall Survival miRNA signature and Distant Disease-Free Survival (DDFS) signature. Overall survival (OS) of TNBC patients of 50 yrs and younger patients due to differentially expressed miRNAs in the three classes. (**A**) Heat map representing miRNA profiles of 75 tumor samples using average linkage clustering and Spearman Rank method as distance metrics. Bar above the dendrogram identifies 39 high risk samples shown in orange and 36 low risk cases in yellow. Samples are shown in columns, miRNAs in rows. Heat map represents relative miRNA expression as indicated in the blue to red key bar at the top. (**B**) Hazard ratios of protective and risky miRNAs. (**C**) Overall Survival miRNAs signature. Distant Disease-Free Survival (DDFS) miRNA signature of 50 yrs and younger. (**D**) Heat map representing miRNA profiles of 75 tumor samples using average linkage clustering and Spearman Rank method as distance metrics. Bar above the dendrogram identifies 37 high risk samples shown in orange and 38 low risk cases in yellow. Samples are shown in columns, miRNAs in rows. Heat map represent relative miRNA expression as indicated in the key bar at the top. (**E**) miRNAs predicting protection from or susceptibility to early recurrence. (**F**) Kaplan-Meier estimates of DDFS according to the seven-miRNA signature.

We investigated associations between miRNA expression levels and survival, for the entire TNBC cohort, as well as for the >50 years of age patients (50+, mostly post-menopausal) and the 50 years and under patients (50−, mostly premenopausal, but including some patients who had hysterectomies). Here, we present a detailed analysis of the 50− cohort, while further investigations for the 50+ subset are ongoing. For the 50− patients, the median follow-up was 79 months (range 9–194 mo), the median age was 43 (range 20–50 yrs). Censoring occurred at the date of death from any causes (overall survival, OS), first evidence of distant recurrence (distant-disease free survival, DDFS) or at time of the last known follow-up, whichever occurred first. All expressed miRNAs (n = 133) in tumor samples were considered. For both OS and DDFS, we used Cox proportional hazards models and identified sets of miRNAs that are significantly related to outcomes. We then performed permutation tests in which the times and censoring indicators were randomly permuted among samples. Permutation P values for significant miRNAs were computed based on 10,000 random permutations. Hazard ratios (HR) were computed for a 2-fold change in the miRNA expression level. 4 miRNAs were significantly associated with OS, as determined by univariate and multivariate analysis. Of these, 3 were up-regulated and 1 down-regulated in the normal *vs* tumor comparison (Fig. 2A). ‘Protective’ miRNAs were defined as those associated with an HR (from univariate Cox regression analysis) of less than one (HR<1); ‘risk-associated’ miRNAs were defined as those associated with an HR greater than one (HR>1). Up-regulation of miR-16 (HR = 0.87, 95% CI = 0.79–0.94), miR-155 (HR = 0.728, 95% CI = 0.57–0.92), or miR-374a (HR = 0.85, 95% CI = 0.72–0.99) correlated with better prognosis (protective); down-regulation of miR-125b (HR = 1.355, 95% CI = 1.03–1.79) correlated with a worse prognosis (risk-associated) (Fig. 2B). All tumors were classified into high- or low-risk groups according to their risk-score (see Materials and Methods). The Kaplan-Meier OS graph, according to the combined 4 miR signature in Fig. 2C, shows divergent OS curves (P = 0.05). The median OS for the high *vs* low risk miR signature was 69 *vs* 83 mo, HR 2.18 95% CI = 0.97–4.84 P = 0.05, indicating association between expression of the signature miRNAs and OS.

For DDFS the median follow-up was 75 months (range 6–194 mo.). 7 miRNAs were significantly associated with DDFS, as determined by univariate and multivariate analysis. These 7 miRNAs were significantly differentially expressed in the normal *vs* tumor comparison, 4 up-regulated and 3 down-regulated in Fig. 2D. In Fig. 2E we show 3 “risk-associated” (miR-125b, 655, 421) and 4 “protective” miRNAs (miR-16, 374a/b, 497). The 7 miRNAs signature illustrated by Kaplan-Meier graph (Fig. 2F) shows tumors with a high-risk miR signature associated with a lower median DDFS than tumors with a low-risk miR signature (51 *vs* 81 mo) (the HR of high-risk *vs* low-risk signatures, 3.46, 95% CI = 1.35–8.85 P<0.01), showing a strong association between this miR signature and DDFS.

### mRNA expression profiles and predicted correlations with expression of specific miRNAs

**Figure 3:**
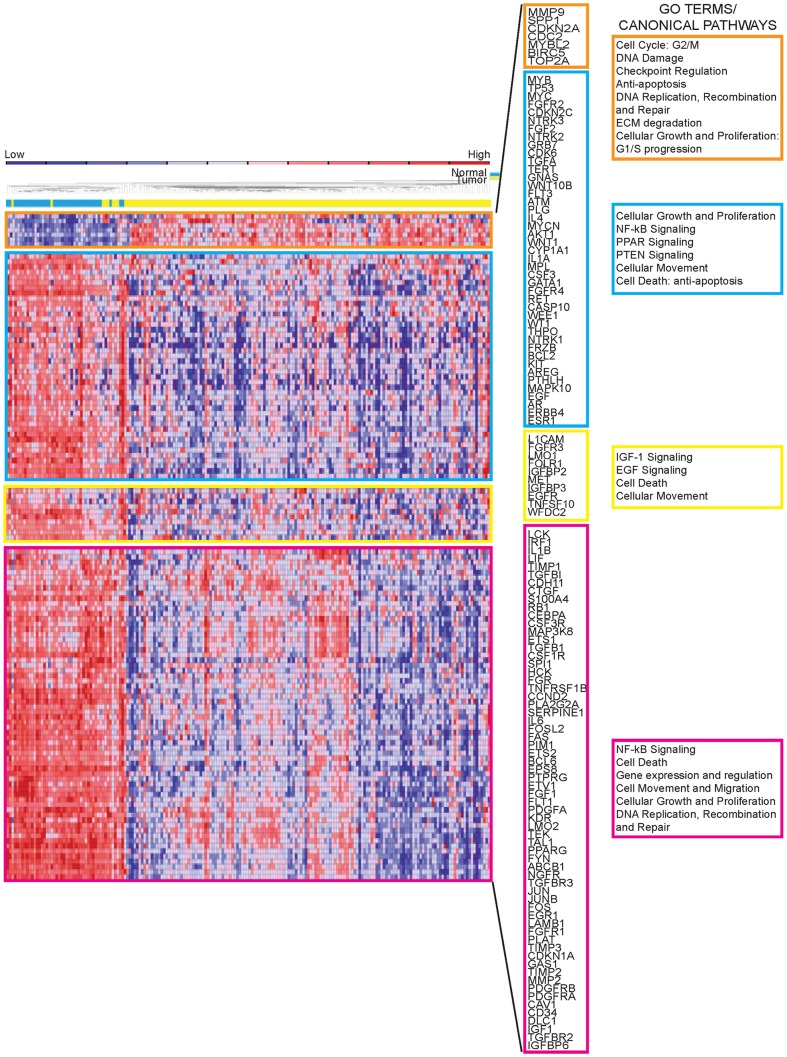
Comparison of mRNA expression profiles of normal *vs* tumor RNAs. The heat maps represent hierarchical clustering of differentially expressed genes in normal and tumor-derived RNAs. mRNA profiles are clustered in 4 different subgroups (orange, blue, yellow, pink) defined by the mRNA expression patterns. Overlapping Gene Ontology terms for top canonical pathways represented by the differentially expressed genes in each subgroup, as determined by IPA-ingenuity software, are shown on the right for each of the normal/tumor comparison-defined subgroups.

The nanoString GX Human mRNA Cancer Reference panel was used to profile expression in 158 tumors, 40 adjacent normal tissues and 54 lymph node mets. The profiles discriminated non-tumor tissue from TN tumors and mets. Hierarchical clustering represented in the heat maps (Fig. 3) show 124 mRNAs differentially expressed in TNBC relative to adjacent non-tumor breast samples (Table S4). The mRNA expression profiling resulted in clustering of the TNBCs into 4 molecular subgroups with different gene expression signatures (Fig. 3). For each cluster of genes we performed functional enrichment assessments of potentially perturbed pathways using the IPA-Ingenuity software. To find links between the prognostic signature miRNAs, their putative target genes and interaction pathways, we performed target prediction analysis and then calculated the Pearson correlation index, focusing on inversely expressed miRNA∶mRNA pairs. For each miRNA we selected the top anti-correlated genes (Table 1).

**Table 1:** Correlation of miRNAs and inversely expressed genes.

miRNA Expression		Gene Expression		microRNA∶mRNA
miRNA	logFC	Gene Symbol	logFC	Expression Correlation
hsa-miR-16	5.73	*CCND1*	−0.48	−0.13
		*MYB*	−1.54	−0.14
		*FGFR2*	−1.31	−0.16
		*FGF2*	−2.27	−0.34
		*NTRK2*	−3.38	−0.35
		*CDK6*	−0.70	−0.32
		*FLT3*	−1.31	−0.25
		*RET*	−1.47	−0.39
		*WEE1*	−1.53	−0.47
		*WT1*	−0.95	−0.38
		*BCL2*	−1.88	−0.36
		*EGFR*	−1.99	−0.37
		*CCND2*	−2.00	−0.31
		*PIM1*	−1.34	−0.31
		*ETV1*	−1.75	−0.48
		*KDR*	−1.66	−0.37
		*TGFBR3*	−3.63	−0.41
		*JUN*	−2.09	−0.37
		*FGFR1*	−2.34	−0.43
		*IGF1*	−3.34	−0.45
hsa-miR-125	−2.03	*CDKN2A*	0.75	−0.18
hsa-miR-374a	2.83	*CCND1*	−0.48	−0.15
		*AKT1*	−1.28	−0.36
		*ATM*	−1.17	−0.36
		*BCL2*	−1.88	−0.22
		*CDK6*	−0.70	−0.27
		*FGFR2*	−1.31	−0.10
		*IL1A*	−1.43	−0.34
		*NTRK2*	−3.38	−0.21
		*TGFA*	−0.85	−0.21
		*ETS2*	−1.95	−0.27
		*FAS*	−1.27	−0.26
		*FOS*	−3.22	−0.24
		*GAS1*	−2.07	−0.18
		*MAP3K8*	−1.45	−0.21
		*IGFBP3*	−1.08	−0.15
		*MET*	−1.54	−0.14
hsa-miR-374b	1.92	*CCND1*	−0.48	−0.16
		*AKT1*	−1.28	−0.43
		*ATM*	−1.17	−0.37
		*BCL2*	−1.88	−0.30
		*CDK6*	−0.70	−0.27
		*FGFR2*	−1.31	−0.16
		*IL1A*	−1.43	−0.35
		*NTRK2*	−3.38	−0.32
		*TGFA*	−0.85	−0.21
		*ETS2*	−1.95	−0.42
		*FAS*	−1.27	−0.36
		*FOS*	−3.22	−0.40
		*GAS1*	−2.07	−0.32
		*MAP3K8*	−1.45	−0.29
		*IGFBP3*	−1.08	−0.31
		*MET*	−1.54	−0.25
hsa-miR-421	3.36	*ATM*	−1.17	−0.14
		*CASP10*	−1.34	−0.26
		*CDK6*	−0.70	−0.21
		*TP53*	−0.79	−0.23
		*ETS1*	−0.99	−0.25
		*FOSL2*	−1.20	−0.27
		*IGF1*	−3.34	−0.45
		*LCK*	−0.49	−0.16
		*PDGFRA*	−2.41	−0.48
		*PDGFRB*	−2.12	−0.45
		*SERPINE1*	−1.14	−0.24
		*TIMP2*	−1.83	−0.40
hsa-miR-655	−1.56	*CDKN2A*	0.75	−0.07

The degree of anti-correlation among the mRNA–miR pairs is calculated by Pearson correlation. The predicted targeted-anti-correlated genes are shown.

*Molecular Subgroup 1 (ORANGE)* includes 7 mRNAs, for *SPP1*, *MMP9*, *MYBL2*, *BIRC5*, *TOP2A*, *CDC2*, *CDKN2A* genes, that are over-expressed in tumor compared to normal; these genes are involved in critical biological functions: *CDC2* (logFC 1.2) in regulation of G1 progression, G1/S and G2/M transition [20]; *MYBL2* (logFC 2.3) in regulation of S-phase and activation/repression activities, such as activation of CDC2 protein, cyclinD1, and insulin-like growth factor-binding proteins, and in DNA damage response; BIRC5 gene product (logFC 1.9) in apoptosis inhibition; TOP2A protein (logFC 1.8) in response to anthracycline chemotherapy [21]; MMP9 protein (logFC 1.5) in invasion and proliferation (up-regulated in tumors and mets) and in DNA replication, recombination and repair. Within this set of genes the correlation coefficient strongly links the ‘risk-associated’ miR-125b and miR-655 with the *CDKN2A* gene (logFC 2.6), predicted target of these two miRNAs.

*Molecular subgroup 2 (BLUE)* includes 43 mRNAs down-regulated in the tumors. The top gene ontologies for this molecular subgroup are enriched in NF-kB, PPAR and PTEN signaling pathways. The cellular growth associated genes have these expression fold changes: *BCL2* (logFC −1.8), *EGF* (logFC −1.9), *ERBB4* (logFC −2.6), *ESR1* (logFC −3.4), *IL1A* (logFC −1.3) and *FGFR2* (logFC −0.8), *WT1* (logFC −1.1), *MYC* (logFC −1.4), *FGF2* (logFC −2.5); *AKT1* (logFC −1.3), *CASP10* (logFC −1.4), involved in cell movement, cell death and cell development. The Pearson correlation links the ‘protective’ miR-16 to these genes and their pathways; 10 of its top predicted targets belong to this group (Table S2). Other ‘protective’ miRNAs, miR-374a/b and ‘risk-associated’ miR-421 are highly anti-correlated with important genes of this group, which are also their predicted targets, *NTRK2* (logFC −2.9) a regulator of cell proliferation, *ATM* (logFC −1.3) involved in recognizing damaged DNA strands, *BCL2* and *AKT1*, implicated in cell death.

*The third molecular subgroup (YELLOW)* is represented by 10 deregulated mRNAs. These transcripts are enriched for gene ontologies involving growth factors (IGF-1 and EGF signaling). This group is also enriched in expression of growth factor receptor *MET* (logFC −1.4), which ensures cell survival, and *L1CAM* (logFC −0.7) and *IGFBP3* (logFC −1.2), which are involved in cell movement. ‘Protective’ miR-16 is predicted to target *EGFR* and they are negatively correlated, miR-374a/b are anti-correlated with predicted targets *IGFBP3* and *MET* (Table 1).

*The fourth and largest subgroup (PINK)* is characterized by the expression of 64 mRNAs, with NF-kB signaling as the top canonical pathway. The gene ontologies are enriched in components, pathways or functions involved in: cell death, growth and proliferation, represented by *TIMP1* (logFC −0.8), *TIMP2* (logFC −1.7), *CDKN1A* (logFC −1.7), *CCND2* (logFC −1.9), *MAP3K8* (logFC −1.6); cell movement, represented by *CAV1* (logFC −2.8), a potential growth suppressor in breast cancer. Other top molecular and cellular functions are: cell migration, represented by *LAMB1* (logFC −1.6) a positive regulator of migration, and gene expression; cell cycle regulation, represented by *JUN* (logFC −2.2); transcriptional regulation represented by *CEBPA* (logFC −1.8) a promoter binding co-factor and positive regulator of transcription. The Pearson correlation and target predictions link to this group miR-16, miR-374a/b and miR-421.

We note that the ESR1 probe is in the nanoString cancer mRNA panel and very low expression of this mRNA was detected in 7 TNBCs, as shown in Fig. 3, molecular subgroup 2 (blue); the ESR1 protein was not expressed in these tumors, as determined by immunohistochemical analyses, a basis upon which the tumors were selected for this study.

### mRNAs dysregulated in mets

**Figure 4:**
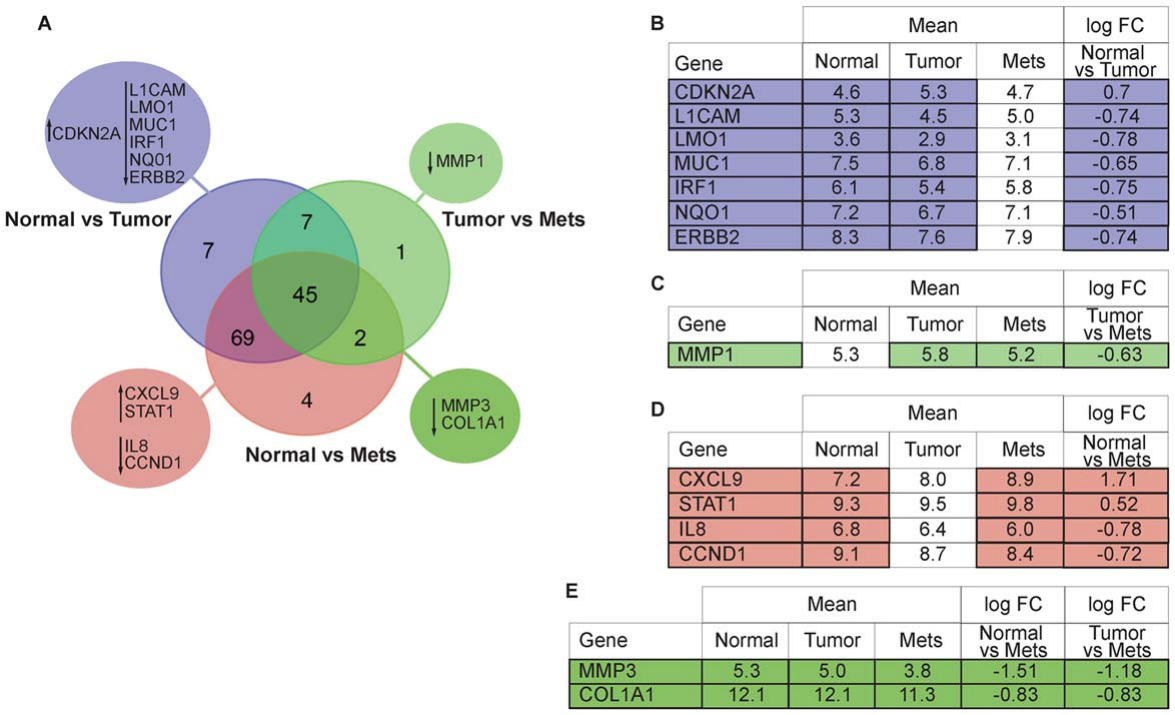
Venn diagram and associated table describing mRNA expression profile patterns in the three classes. Venn diagram (**A**) representing differentially expressed mRNAs observed in the comparison among the three classes (normal, primary tumor, metastatic lesion) for patients of 50 yrs and younger, up or down facing arrow indicate the expression level change. Tables show: the 7 mRNAs identified by the comparison between Normal *vs* Tumor (**B**); 1 mRNA differentially expressed only in Tumor vs Mets (**C**); 4 mRNAs differentially expressed in Normal vs Mets (**D**); 2 mRNAs commonly deregulated in the comparisons Tumor vs Mets and Normal vs Mets (**E**).

Hierarchical clustering of a subset composed of 40 normal and 50 regional lymph node mets RNAs (Fig. S4) identified 120 mRNAs that were significantly differentially expressed, 112 mRNAs up- and 8 down-regulated in mets. For patients with a median age of 43 years (range 20–50 yrs) the mRNA profiles are summarized in the Venn diagrams in Fig. 4 A. In Fig. 4 B we show the mRNAs deregulated in the normal *vs* tumor comparison, although to find possible links between genes and the previously shown miRNA “metastatic signature” we focused our attention on the mets comparisons. In Fig. 4 C we report the only significantly differentially expressed gene, characteristic of the comparison: tumor *vs* mets (*MMP1*, logFC −0.6). 4 genes were notable in normal *vs* mets (Fig. 4 D): *CXCL9* (logFC 1.7), *IL8* (logFC −0.8), *STAT1* (logFC 0.5), *CCND1* (logFC −0.7). For *CCND1*, here down-regulated, studies showing both positive and negative associations with prognosis have been reported. *CCND1* gene amplification has been related to poor disease outcome in ER-positive cancers, but other studies have correlated cyclin D1 protein expression with both better and worse prognosis. It is known that amplifications of *CCND1* and *MYC* frequently occur together in breast cancer [22]; accordingly, we observed its down-regulation coupled with *MYC* down-regulation (logFC −1.2). In the normal *vs* mets comparison, the expression trend for 2 genes was intriguing: up-regulation of *CXCL9* (logFC 1.7) and down-regulation of *IL8* (logFC −0.8). The ability of CXCL9 protein (migratory monokine-induced by interferon-γ) to modulate host cell infiltration and tumor behavior is known; CXCL9 protein expression also results in smaller tumors and decreased rate of tumor metastasis. Recent studies are focusing on the metastatic process after the “nesting” phase [23], [24]; it is believed that once the tumor has nested in another site, the metastatic markers are turned off by the tumor microenvironment, in favor of markers more favorable to tumor proliferation. Similarly, interleukin-8 (IL-8), discovered as a chemotactic factor for leukocytes, has recently been shown to contribute to cancer progression through its functions as a mitogenic, angiogenic, and motogenic factor [25]. Elevated IL-8 levels predict early metastatic spread of breast cancer, may be negatively correlated with ER-status and expressed preferentially in invasive cancer cells [26]. The *IL8* down-regulation observed in the normal *vs* mets comparison may reflect reprogramming in absence of the need of metastatic cells to extravasate after reaching the metastatic site in the lymph nodes. Fig. 4 E shows the mRNAs commonly deregulated in the normal *vs* mets and tumor *vs* mets comparisons (*MMP3* logFC −1.2; *COL1A1* logFC −0.8).

## Discussion

Stratification of TNBC into subclasses using new markers will identify new screening methods, prognostic factors, methodologies and perhaps targets for personalized therapies. Several recent studies have correlated miRNA expression with outcomes in TNBC using microarray or other high throughput technologies. mRNA expression profiles that sub-classify TNBCs have also been reported in association with investigations of outcome, new molecular pathways and possible chemotherapy alternatives [27], [28]. We have carried out a large-scale analysis of miRNA and cancer-focused mRNA expression in normal, triple negative tumor and associated metastatic tissues.

We first profiled miRNAs expressed in specific tissue classes for the entire cohort. Subsequent focus on the 50 and under subset allowed identification of 2 miRNA signatures, distinguishing ‘protective’ and ‘risky’ prognostic miRNAs; these prognostic signatures require validation on an independent group of TNBC patients and this work is in progress. The primary tumor mRNA expression profiles of the entire cohort clustered into 4 molecular subgroups. Through miRNA target prediction and use of IPA software, we determined that the prognostic signature miRNAs were connected with the 4 molecular subclasses, in which they are predicted to target many transcripts and participate in control of their canonical signal pathways.

### The miRNA profile

13 miRNAs (Fig. 1A) differentially expressed in the normal *vs* tumor comparison, represent a strictly tumor-specific pool for which IPA ingenuity software identified observed targets and signal pathways involved; examples are Integrin, protein kinase A, Thrombin, Phospholipase C, FAK, RhoA, ILK, and CAMP signaling, proliferation/apoptosis related and thus expressed in the tumor microenvironment [29].

In tumor *vs* mets and normal *vs* mets comparisons, a total of 6 miRNAs were differentially expressed in the mets tissues, miR-424, miR-125a-5P, miR-627, miR-579, Let-7g, miR-101. Considering only the known targets of these miRNAs we can track the pathways involved, integrin, VEGF, ILK, tight junction signaling and NRF2-mediated oxidative stress response, pathways involved in the metastasis process, cell survival in the vasculature, and extravasation from the blood stream. These miRNAs and associated signal pathways confirm the metastatic origin of the lymph node mets RNAs. Among the 6 mets-associated miRNAs, miR-424 is interesting as the only one differentially expressed in the mets relative to primary tumors. Aberrant miR-424 expression has been observed in other cancer types as well, an indication of its potentially pivotal role in the development of metastases [30]. IPA software correlates miR-424 (together with let-7g) to the NRF2-mediated oxidative stress response pathway, necessary for survival of cancer cells in the vasculature during the metastasis process, and thus this miRNA is expressed in the primary and regional nodal metastases.

miR-125a, the only miRNA significantly dysregulated only in the normal *vs* mets comparison may function as a tumor suppressor in breast cancer and in breast cancer cell lines, as its over-expression inhibits proliferation, motility and invasiveness, cell migration and promotion of apoptosis. The 15 miRNAs commonly dysregulated across the three tissues classes presented in the center of the Venn diagram (Fig. 1A) are the most dysregulated of the entire panel with fold changes >3. Some were known to be involved in cancer; others such as miR-26b, 548g, and 660 show large fold changes but associations with TNBC and metastasis were not previously known.

Using univariate and multivariate Cox analysis we investigated the correlation of differentially expressed miRNAs in TNBC samples with OS and DDFS, in a subset of cases (a somewhat homogeneous cohort of patients aged 20–50 yrs) and observed 4 miRNA and 7 miRNA signatures prognostic for OS and DDFS, respectively, with ‘protective’ miR-16, 374a and ‘risk-associated’ 125b included in both signatures. In invasive breast cancers, association between down-regulation of miR-125b and poor survival has been reported [31], and miR-125b over-expression can confer resistance of breast cancer cells to paclitaxel [32]. From analysis of the mRNA profile, we also predict that the important tumor suppressor *CDKN2A* is a target of miR-125b and miR-374a has been related to survival in lung cancer [33]; in Table S4 we report several anti-correlated, predicted targets that could explain its ‘protective’ action: *ATM*, *GADD45A*, *CCND1* have key DNA damage response roles; Ets2, Met, Fos are fundamental proteins in cancer signaling so their down-regulation by over-expression of miR-374a is supportive of its ‘protective’ capacity. miR-16 can act as a tumor suppressor through pathways involving *BCL2* and *CCND1*, validated targets (here also anti-correlated). Other potential targets of this miR are *MYB*, *JUN*, *CCND2*, *WEE1*, all anti-correlated in our profile, and their down-regulation would be consistent with the better survival for patients over-expressing miR-16. The last miRNA predicting OS is miR-155 defined as ‘protective’. So far nothing has been reported concerning a protective role for miR-155; its up-regulation has been reported in several types of solid tumor, correlating with poor prognosis and promoting cancer growth and cell survival [34], [35], [36]. However, it has been reported that the location of tumor cells over-expressing miR-155 is a critical factor: in mammary fat pads miR-155 prevents tumor dissemination, reduces aggressiveness by preventing epithelial-to-mesenchymal transition (EMT), suppressing the expression of the transcription factor TCF4. McInnes et al. [37] recently described a forward loop that shows how miR-155 may target the *SATB1* oncogene. *SATB1* is not only a direct target of FOXP3 repression, but FOXP3 also induces miR-155 over-expression, which specifically targets the 3′-UTR of *SATB1* to further down-regulate its expression. Clearly mechanistic biological studies are needed to confirm expression of this miRNA as protective in TNBCs.

Seven miRNAs were prognostic for DDFS. miR-497, a down-regulated member of the miR-16 family, has a ‘protective’ function. It was reported to be down-regulated in breast cancer, partially due to DNA methylation [38], is known to directly target *BCL2* and modulate drug resistance. As for the ‘risk-associated’ miR-655, little has been reported other than that its down-regulation is strictly anti-correlated with expression of its predicted target *CDKN2A*. miR-374b, defined as ‘protective,’ and miR-421 ‘risk-associated’, form an “miRNA cluster” (miR-374b/421). Contrasting findings have been observed in different cancer models. miR-374b can suppress MYC expression by targeting its 3′-UTR, miR-421 can suppress ATM expression [39] but this is the first report associating this cluster with survival. Identification of outcome-associated miRNA signatures suggests that patients with TNBCs with the high-risk miRNA signature might benefit from aggressive adjuvant therapy.

### The Cancer-focused mRNA profile

The small amount of RNA available necessitated assessment of mRNA expression of a limited set of genes represented by the nanoString Cancer Reference panel, a limitation in this molecular profiling study. Nevertheless, we observed dysregulated expression profiles among these 230 mRNAs in TNBC. The 124 mRNAs that were significantly deregulated clustered into 4 color-coded molecular subgroups representing the most specifically perturbed pathways in TNBC.

Gene expression clusters and IPA Ingenuity analysis gave an overview of critical pathways involved in this TN cohort. As expected in a tumor cohort (orange subgroup), processes such as cell cycle, DNA damage check point, anti-apoptosis, ECM degradation, cell growth and proliferation are turned on; more surprising was the down-regulation of known cancer-associated pathways detected in the other three subgroups. The oncogenes *MET*, *MYC*, *BCL2* (yellow and blue subgroups) were down-regulated in normal *vs* tumor. We observed that the miRNAs directly targeting these oncogenes were over-expressed in those TNBC subgroups: miR-340 (logFC 1.82) targets *MET*; miR-21 (logFC 2.29) and miR-98 (logFC 0.63) targets *MYC*; miR-15a (logFC 2.24), miR-15b (logFC 1.07), miR-16 (logFC 5.35), miR181a (logFC 1.58), miR-21 are all up-regulated and target *BCL2*. Contributing explanations for these surprising findings may be the dysregulated expression of several transcription factors, negative regulators of the oncogenes. *WT1* down-regulation directly down-modulates MYC and BCL2 expression; similarly, Rb-1 regulates MYC, Ets1 down-regulates MET and CEBPA [40].

Analysis of the mRNA-defined molecular subgroups, together with the OS and DDFS prognostic miRNAs, exploiting the Pearson correlation coefficient, allowed definition of correlations of most of the prognostic miRNAs (excepting miR-155 and 497) with their predicted gene targets lending strong support to the results. Though these miRNAs are reportedly involved in breast cancer, none has previously been associated with OS or DDFS. Only two, miR-125b and -16, have been shown to contribute to drug resistance in breast cancer; further investigation of the signature miRNAs is ongoing.

In comparisons of mRNA expression patterns in the three tissue classes, the metastatic tissue group gave interesting results. Up-regulation of *STAT1* in mets agrees with findings of several previous studies: its over-expression can confer a more malignant and therapy-resistant phenotype; STAT1 over-expressing TNBCs are more aggressive; increased STAT1 protein abundance might also enhance the invasiveness of breast cancer cells [41]. Also *CCND1* expression, reportedly down-regulated in breast cancer, was confirmed in our profile, through the down-regulation of *MYC*, reported to be coupled with *CCND1* expression [42].

Interestingly, our novel findings concerning expression levels of *CXCL9*, *IL8*, *MMP1*, *MMP3* and *COL1A1* (Fig. 4B) are the converse of previously reported results: CXCL9 protein can modulate host cell infiltration and tumor behavior. The up-regulation of *CXCL9* and down-regulation of *IL8* observed in our study are indirectly confirmed in our profile by considering expression levels of their upstream regulators. Expression of *CXCL9* and *IL8* are regulated by Cyclooxygenase-2 (COX-2) protein, which is expressed at extremely low levels in this set of mets (data not shown). COX-2 inhibition increases release of CXCL9 and CXCL10 proteins from breast cancer cells [43] and induces IL-8 down-regulation. *MMP1* and *MMP3* genes are usually found to be up-regulated in mets. Here these genes are down-regulated, with the same trend observed for the MMP upstream regulators. Down-regulation of the transcription factor Ets-1 directly down-regulates expression of MMP1, with *ETS1* expression usually up-regulated together with *MMP1* and *MMP9*; *WT1* expression, an *ETS1* regulator, is down in our normal *vs* mets comparison as is *ETS1* (data not shown). Increased expression of *MYB* found in the tumor *vs* mets comparison (logFC 1.2) may also contribute to *MMP1* down-regulation; Myb protein is known to up-regulate cathepsin D and MMP9 and down-regulate MMP1 [44]. Collagen 1A1 (COL1A1) protein, the major component of Extracellular Matrix (ECM), is included in the gene signature of breast metastasis [45] and is usually up-regulated in mets. In our mets profile *COL1A1* is down-regulated together with *S100A4* and *MYB*, two of its regulators. Altogether these findings, up-regulation of *CXCL9* and down-regulation of *IL8*, MMPs and *COL1A1* in the mets cohort, suggest that over-expression of these proteins, while needed for “extra-vasation” and perhaps for “nesting” of the cancer cells after intravasation, may be down-regulated after the tumor has become established in another site, in favor of genes and gene products promoting tumor proliferation.

In conclusion, in TNBC, integrated miRNA-mRNA profiling can distinguish the different breast tissue “stages”: tumor adjacent, tumor and lymph node metastasis. miRNA signatures can be prognostic markers for OS and DDFS, suggesting that therapies tailored to these markers may contribute to improved survival.

mRNA profiling also emphasized the heterogeneity of TNBC, as exemplified by the varying signal pathways driving tumors and metastasis of the mRNA-defined subgroups.

## Materials and Methods

### The TNBC tissues

An IRB-approved OSU protocol for this research linked clinical features, treatment and outcome data of breast cancer patients in the OSU National Comprehensive Cancer Network breast cancer database/tumor registry with archrival breast cancer pathology specimens stored in the OSU Tissue Archive Service using the Information Warehouse at OSUMC to serve as “honest broker” and provided de-identified clinic-pathological information. From 1995–2005, a cohort of 365 consecutive triple negative localized breast cancer patients were identified. After pathology review for tumors with sufficient sample for study, the only selection criterion, 173 paraffin blocks for TNBCs were identified for preparation of a tissue microarray and cores for RNA preparation, with the characteristics shown in the demographics summary in Table 2. For 50 of the primary tumors there were also fixed matched lymph node metastatic lesions and for another 4 patients there were only lymph node lesions.

**Table 2:** Demographic features of 173 TNBC cases.

Characteristic		All (173)	>50 yr (87)	<50 yr (86)
**Race**	Caucasian	153	82	71
	African American	16	4	12
	Other	4	1	3
**Menopause status**	Pre-menopausal	64	3	61
	Post-menopausal	103	81	22
	Unknown	6	3	3
**Grade**	I	2	0	2
	II	15	10	5
	III	148	76	72
	IV	2	0	2
	Unknown	6	1	5
**Basal**	Yes	78	42	36
	No	95	45	50
**Lymph node metastases**	Positive	102	72	30
	Negative	62	12	50
	Unknown	9	3	6
**Age at diagnosis**	< = 40	34		
	41–50	52		
	> = 51	87		
**Death**	Yes	59	30	29
	No	114	57	57
* **Recurrence**	No	126	67	59
	Yes	47	20	27
* **Type of 1st recurrence**	In situ	1	0	1
	Local/Regional	3	1	2
	Distant	35	15	20
	Type unknown	8	4	4

* Does not include the cases that were never disease-free, those unknown if ever disease-free and those with missing recurrence information. The demographic features of the original 365 TNBCs were nearly identical to those shown above for the 173 TNBCs analyzed.

### RNA preparation

RNA was isolated from formalin-fixed paraffin-embedded tissue of 165 tumor, 59 tumor-associated, adjacent normal and 54 associated lymph node mets tissues, using the Recover ALL kit (Ambion). Due to small amounts of RNA available from the 251 formalin-fixed paraffin-embedded cores, we have used the nanoString nCounter human miRNA expression profiling v1 panel and mRNA cancer panel, which allows profiling from 100 ng of RNA per sample [46]. Data were processed using several normalization strategies, including quantile normalization and normalization to a set of invariant miRNAs [47].

### nanoString nCounter profile analysis

RNAs were processed with the nanoString nCounter system (nanoString, Seattle, Washington, USA) in the Nucleic Acid Shared Resource of The Ohio State University. The miRNA panel detects 664 endogenous miRNAs (with 654 probes), 82 putative viral miRNAs, and five housekeeping transcripts.

For analysis of mRNA expression, the nanoString GX Human mRNA Cancer Reference panel, that includes tags specific for 230 cancer-related mRNAs (http://www.nanostring.com/products/gene_expression_panels.php), was used.

### GO analysis of miRNA targets

The ‘Core Analysis’ function included in the Ingenuity Pathways Analysis (IPA) software (http://www.ingenuity.com/) was used to interpret data in the context of biological processes, pathways and networks. After analysis, generated networks are ordered by scores defining significance. Significance of biofunctions and canonical pathways were tested by the Fisher Exact test. TargetScan (http://www.targetscan.org) was used to identify possible interactions between the deregulated miRNAs and mRNA.

### Data analysis, statistical methods, and figures

Raw expression data were log-transformed and normalized by the quantile method after application of a manufacturer-supplied correction factor for several miRNAs. Data were filtered to exclude relatively invariant features (IQR = 0.5) and features below the detection threshold (defined for each sample by a cutoff corresponding to twice standard deviation of negative control probes plus the means) in at least half of the samples. Using R/Bioconductor and the filtered dataset, linear models for microarray data analysis were employed with a contrast matrix for the following comparisons: normal vs tumor, normal vs mets, tumor vs mets. P values were used to rank miRNAs of interest, and correction for multiple comparisons was done by the Benjamini-Hocheberg method. Correlations were determined using the Pearson correlation coefficient (r). The mean time to first relapse was compared between groups using the rank sum test. Analysis of OS and DDFS, was performed by the Kaplan-Meier method, and comparisons of outcomes among subgroups were performed by using the long-rank test. Two-tailed tests were used for univariate comparisons. For univariate and multivariate analysis of prognostic factors (using tumor grade and age as covariates), a Cox proportional hazard regression model was used. Data processing and analysis were conducted using BRB-ArrayTools [48], the MultiExperiment Viewer [49], and R/BioConductor packages [50].

To investigate the differences among the gene expression profiles detected by the nanoString GX Human mRNA Cancer Reference Kit, we performed hierarchical clustering using the 124 dysregulated genes (P-value<0.05) in the entire Normal versus Tumor samples dataset. Two-dimensional average-linkage hierarchical clustering of a Spearman rank correlation similarity matrix of the primary tumors and normal samples was performed. All gene expression analyses were performed using R software (version 2.13.0). As expected, we identified two distinct patient clusters (Normal and Tumor samples) and four distinct gene clusters designated by different colors (Figure 3). In order to identify these clusters, we set a correlation threshold value between −1 and 1 where any genes paired with correlations greater than that threshold can be considered a cluster, and any genes or clusters with correlations less than that threshold are not. This method is known as “cutting the tree”. Functional classification of each gene cluster was identified by the IPA software (version 8.8). Gene symbols were used as input for the search of biological functions and molecular networks associated with the members of the cluster. Figures and tables were prepared using, Adobe Photoshop and Illustrator.

All fold-changes associated with these analyses are represented in log2 scale (logFC) and we show only data with a P-value of <0.05, considered to indicate statistical significance.

The miRNA and mRNA expression data have been submitted to the Gene Expression Omnibus (GEO) with accession number GSE 41970.

### Validations

To validate the study findings, three approaches were used: first we validated the deregulated miRNAs “*in silico*” using the database published by Farazi et al 2011 [GEO: GSE28884]. From the analysis of this database, based on an Illumina Genome Analyzer IIx deep sequencing platform, we were able to confirm the expression pattern of the 69% of our miRNAs cohort represented among the sequences. Furthermore, we have begun a study of a second TNBC RNA cohort, from 48 FFPE tissues. This second cohort was also profiled by the nanoString nCounter method and the miRNA profiles were analyzed following the criteria for the previous cohort and confirmed the deregulation of 79% of the miRNAs observed to be dysregulated in the current study (unpublished data).

Lastly, in a subset of samples (randomly chosen based on availability of RNAs) we were able to validate the expression levels of a subset of miRNAs (7 differentially expressed miRNAs reported in Fig. 1 plus 2 miRNAs used as normalizers) by TaqMan® qRT-PCR assay. Box plots representing this qRT-PCR based validation are shown in Figure S5. Absence of undetermined values in the Real-Time raw data (not shown) also indicates low levels of RNA degradation in this subset of samples.

### qRT-PCR

cDNA was reverse transcribed from 10 ng of total RNA of each sample using specific miRNA primers from the TaqMan® MicroRNA Assays and reagents from the TaqMan® MicroRNA reverse Transcription Kit, Life Technologies (Grand Island, NY). Subsequently, in the PCR step, PCR products are amplified from cDNA samples using the TaqMan® MicroRNA Assays together with the TaqMan® Universal PCR Master Mix. All the assays were performed in triplicate according to the manufacturer's instructions.

## Supporting Information

Figure S1 Hierarchical clustering of miRNA expression patterns of tumor and normal samples. Heat map representing miRNA profiles of 165 tumor and 59 normal samples using average linkage clustering and Spearman Rank method as distance metrics. Bar above the dendrogram identifies the samples, normal shown in light blue and tumors in yellow. Samples are shown in columns, miRNAs in rows. Heat map from blue to red represent relative miRNA expression as indicated in the key bar at the top.
(PDF)

Figure S2 Clustering of miRNA expression patterns of paired tumor and normal samples. Heat map representing miRNA profiles of 55 tumor and 55 paired normal samples using average linkage clustering and Spearman Rank method as distance metrics. A bar above the dendrogram identifies the samples, tumors shown in yellow and normal light blue. Samples are shown in columns, miRNAs in rows.
(PDF)

Figure S3 Clustering of miRNA expression patterns of normal and metastatic RNAs. Heat map representing miRNA profiles of 54 metastatic and 59 normal samples using average linkage clustering and Spearman Rank method as distance metrics. Samples are shown in columns, miRNAs in rows. A bar above the dendrogram identifies the samples, metastases in purple and normal in light blue.
(PDF)

Figure S4 Comparison of mRNA expression profiles of normal vs metastasis-derived RNAs. The heat map representing expression patterns of 120 mRNAs in 40 normal and 50 metastasis-derived RNAs, using average linkage clustering and Spearman Rank methods as distance metrics. A bar above the dendrogram identifies the samples, Metastatic RNAs shown in purple and normal in light blue.
(PDF)

Figure S5 qRT-PCR validation. Box plots represent expression of 7 deregulated miRNAs in a representative subset of samples of the three tissue groups, assayed by TaqMan® qRT-PCR. Results are represented as 2∧^−ΔCt^ relative expression to RNU6B. Error bars ± s.d., *P<0.05, by two-tailed Student's t test.
(PDF)

Table S1 20 deregulated miRNAs in matched Normal *vs* Tumor samples (**A**) and unmatched (**B**).
(XLSX)

Table S2 103 deregulated miRNAs in the comparison Normal *vs* Mets in the entire cohort.
(XLSX)

Table S3 15 miRNAs differentially expressed across the three tissue group comparisons.
(XLSX)

Table S4 Dysregulated mRNAs in Normal *vs* Tumor comparison in the entire cohort.
(XLSX)
