# Supplementary figures and images for: Integrated MicroRNA and mRNA Signatures Associated with Survival in Triple Negative Breast Cancer
Source: PLoS One. 2013 Feb 6;8(2):e55910. doi: 10.1371/journal.pone.0055910 (PMC3566108; doi:10.1371/journal.pone.0055910)

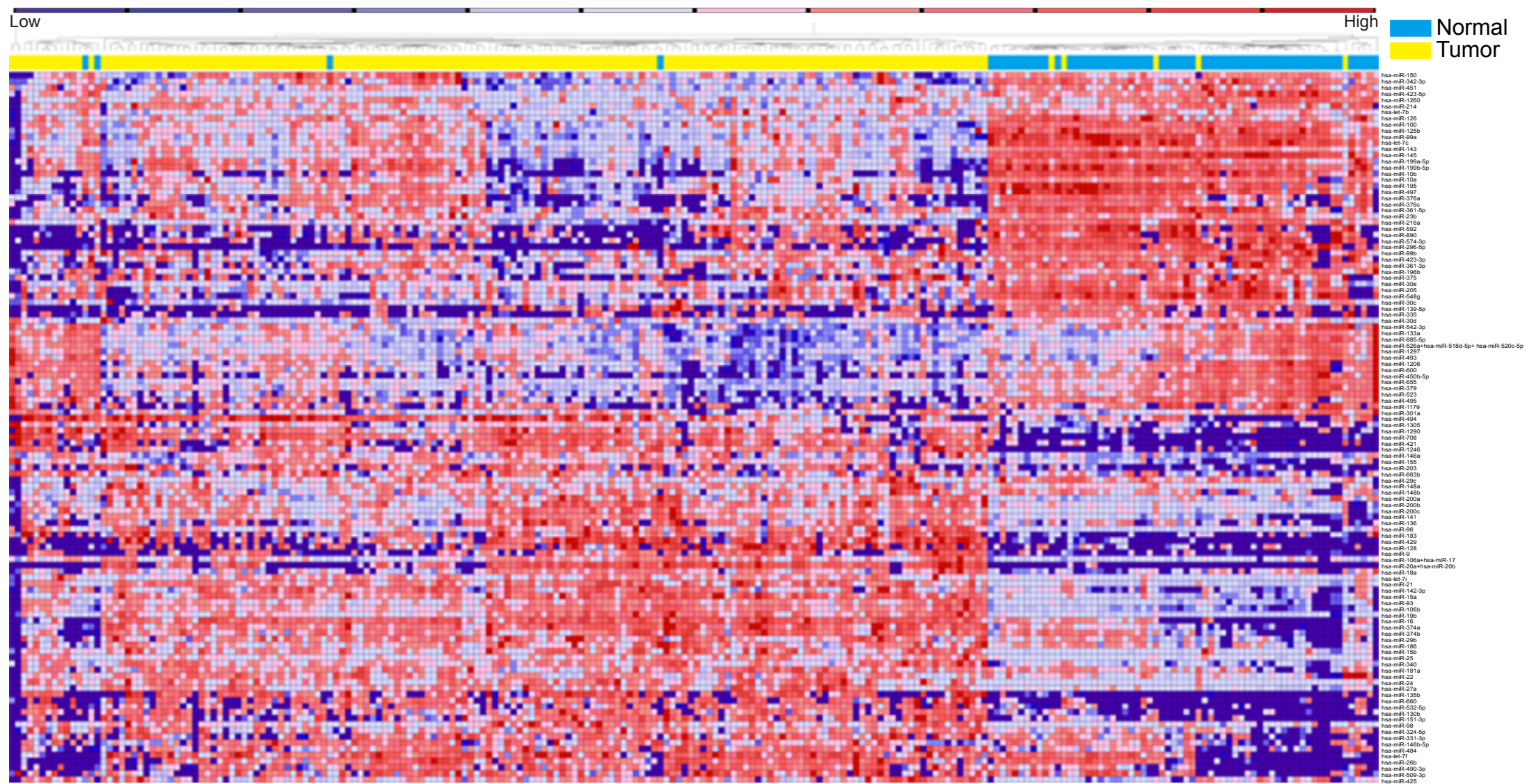

Supplementary Figure 1

Supplement: Figure S1 — Hierarchical clustering of miRNA expression patterns of tumor and normal samples. Heat map representing miRNA profiles of 165 tumor and 59 normal samples using average linkage clustering and Spearman Rank method as distance metrics. Bar above the dendrogram identifies the samples, normal shown in light blue and tumors in yellow. Samples are shown in columns, miRNAs in rows. Heat map from blue to red represent relative miRNA expression as indicated in the key bar at the top. (PDF) [file pone.0055910.s001.pdf]

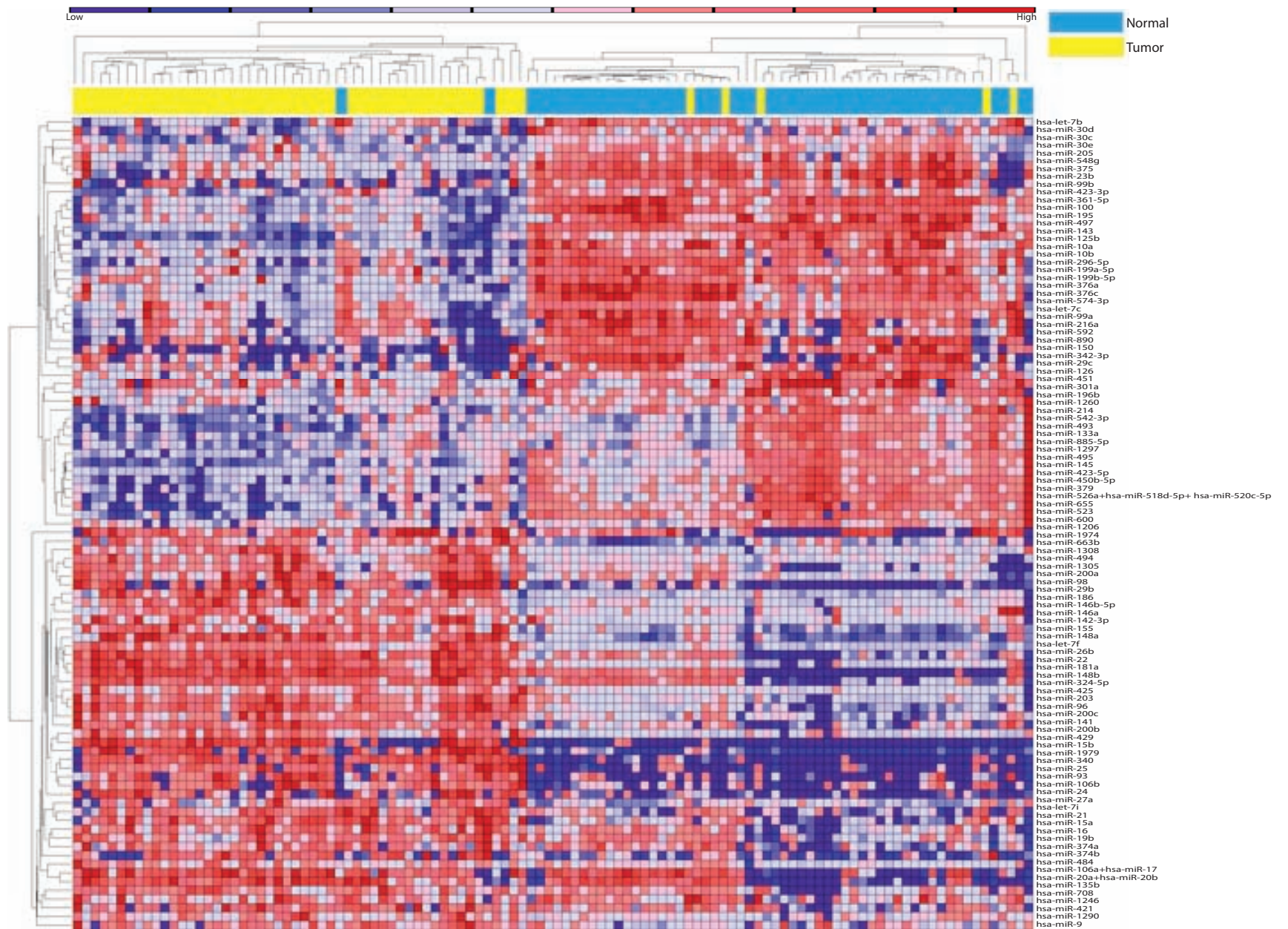

Supplementary Figure 2

Supplement: Figure S2 — Clustering of miRNA expression patterns of paired tumor and normal samples. Heat map representing miRNA profiles of 55 tumor and 55 paired normal samples using average linkage clustering and Spearman Rank method as distance metrics. A bar above the dendrogram identifies the samples, tumors shown in yellow and normal light blue. Samples are shown in columns, miRNAs in rows. (PDF) [file pone.0055910.s002.pdf]

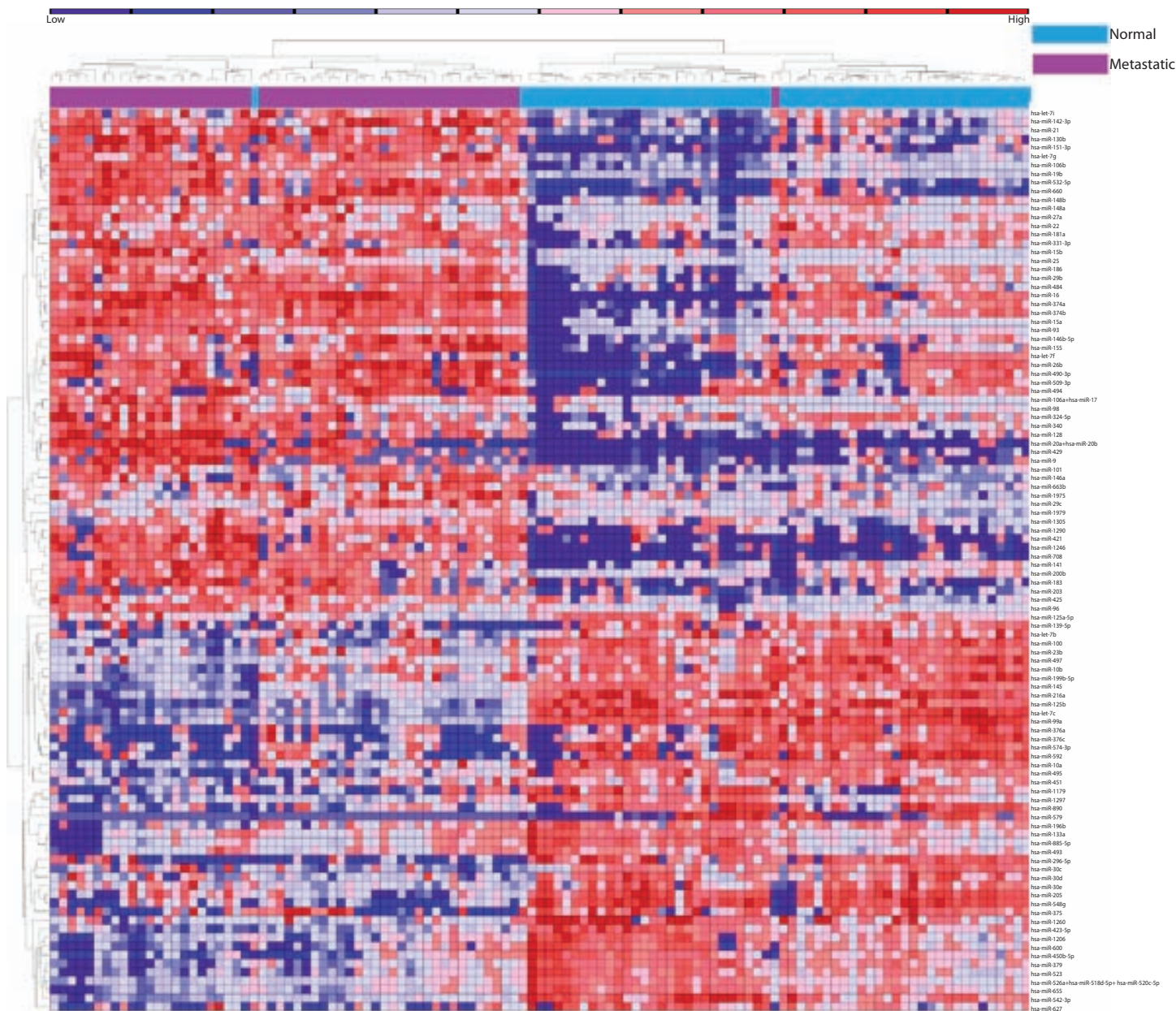

**Supplementary Figure 3**

Supplement: Figure S3 — Clustering of miRNA expression patterns of normal and metastatic RNAs. Heat map representing miRNA profiles of 54 metastatic and 59 normal samples using average linkage clustering and Spearman Rank method as distance metrics. Samples are shown in columns, miRNAs in rows. A bar above the dendrogram identifies the samples, metastases in purple and normal in light blue. (PDF) [file pone.0055910.s003.pdf]

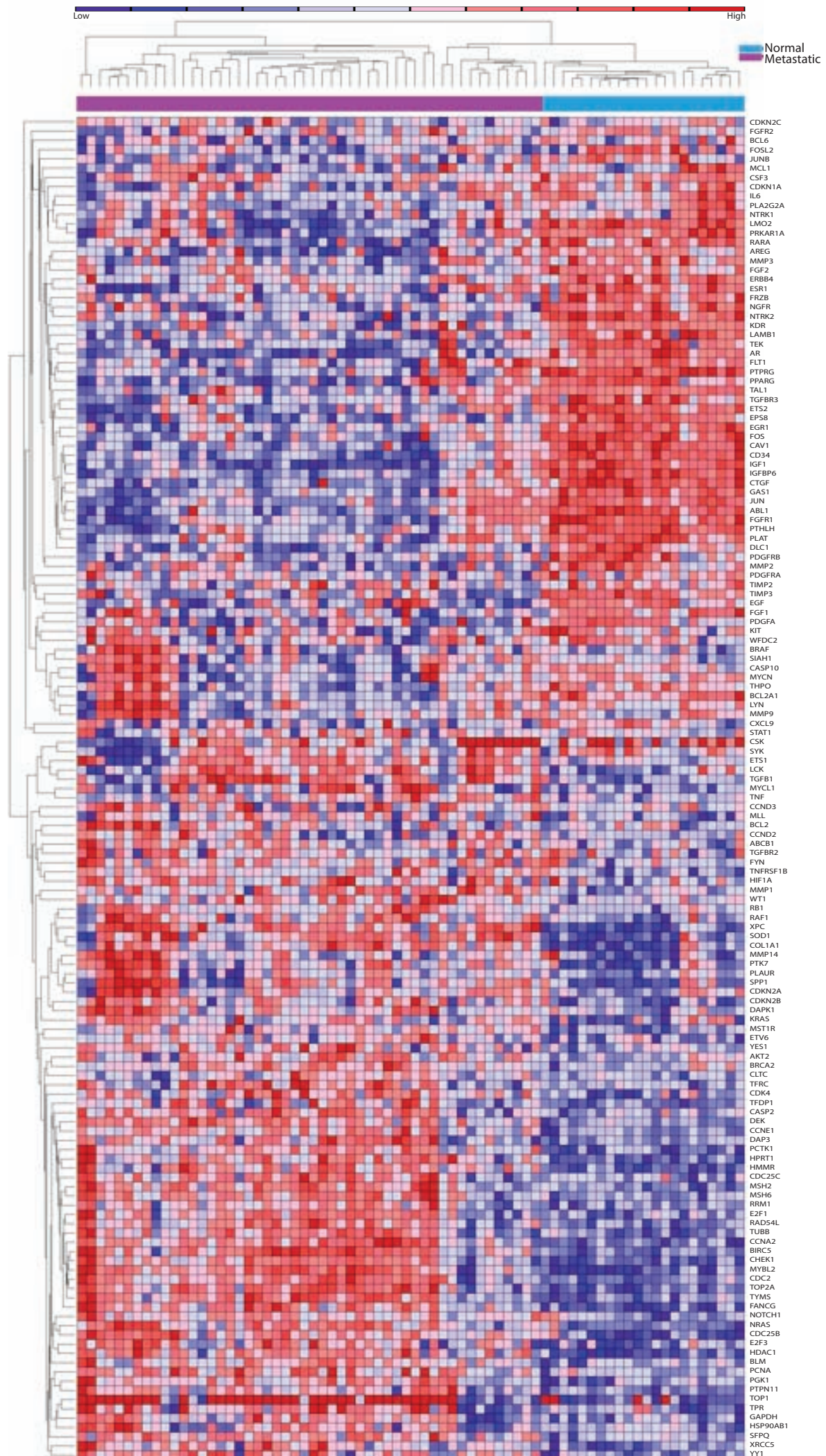

Supplementary Figure 4

Supplement: Figure S4 — Comparison of mRNA expression profiles of normal vs metastasis-derived RNAs. The heat map representing expression patterns of 120 mRNAs in 40 normal and 50 metastasis-derived RNAs, using average linkage clustering and Spearman Rank methods as distance metrics. A bar above the dendrogram identifies the samples, Metastatic RNAs shown in purple and normal in light blue. (PDF) [file pone.0055910.s004.pdf]

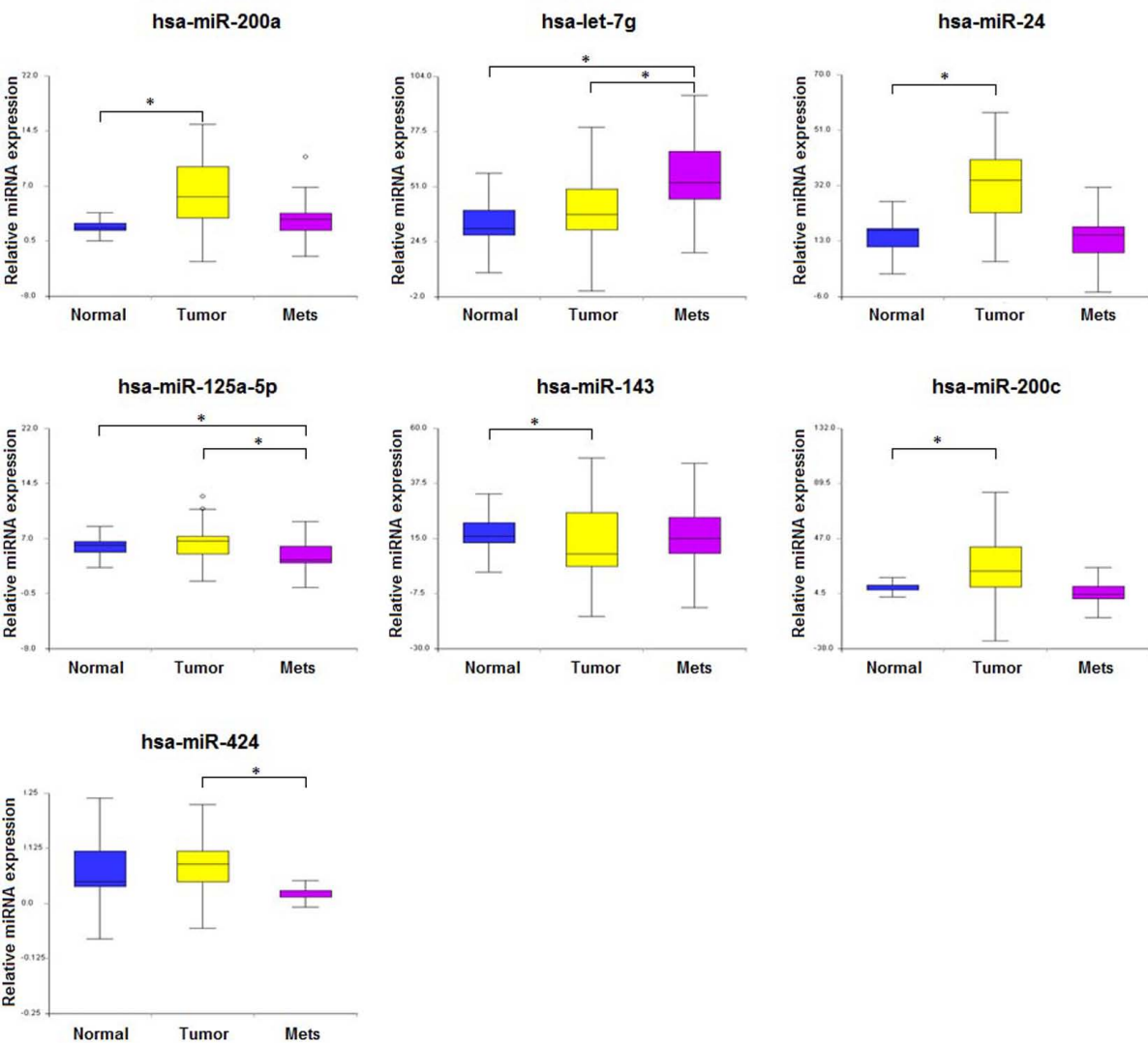

Supplementary Figure 5

Supplement: Figure S5 — qRT-PCR validation. Box plots represent expression of 7 deregulated miRNAs in a representative subset of samples of the three tissue groups, assayed by TaqMan® qRT-PCR. Results are represented as 2∧−ΔCt relative expression to RNU6B. Error bars ± s.d., *P<0.05, by two-tailed Student's t test. (PDF) [file pone.0055910.s005.pdf]
